# Celiac Disease and the Autoimmune Web of Endocrinopathies

**DOI:** 10.7759/cureus.12383

**Published:** 2020-12-30

**Authors:** Ibrahim Sange, Mohamed Wael F Mohamed, Su Aung, Nakul Mereddy, Pousette Hamid

**Affiliations:** 1 Medicine, K. J. Somaiya Medical College and Research Centre, Mumbai, IND; 2 Neurological Surgery, The Royal London Hospital, London, GBR; 3 Medicine/Surgery, University of Medicine, Yangon, MMR; 4 Neurosciences, California Institute of Behavioral Neurosciences & Psychology, Fairfield, USA; 5 Neurology, California Institute of Behavioral Neurosciences & Psychology, Fairfield, USA

**Keywords:** celiac disease, celiac disease and autoimmunity, celiac disease and endocrine system, celiac disease and thyroid, celiac disease and diabetes, celiac disease and glandular autoimmunity

## Abstract

Gluten-sensitive enteropathy or Celiac disease (CD) is a disease that has become very prevalent in most parts of the globe especially in the western world. Resulting from a chaotic interplay between the backgrounds of autoimmunity and genetics, this disorder targets primarily the gastrointestinal tract with ominous extraintestinal counterparts that have a very discrete presentation. Among those counterparts, the one that has been reviewed in this article is the involvement of the endocrine system as concurrence of hormonal disorders with CD possesses numerous challenges that lead to a refractory treatment and a dull prognosis. This review article aims to feature the effect of the CD and endocrine disorders on one another, especially if either of the diseases is asymptomatic, explore the clinical dilemma faced by clinicians in various specialties, and, hence, further pave a path into the importance of rigorous screening and diagnosis.

## Introduction and background

Celiac disease (CD) is an autoimmune disorder of the gastrointestinal tract that occurs in genetically predisposed patients in response to gluten-containing food such as wheat, rye, and barley [1]. In the western population, serological studies were conclusive of a prevalence of almost 1% with a major chunk of the disease population still being undiagnosed [1,2].

In addition to the environmental factors like viral infections and gut microbiota dysbiosis, an obligatory genetic association remains that of the linkage of the disease to human leukocyte antigen (HLA) DQ2/DQ8 [3]. With a minor female predominance, the disease occurs irrespective of age and with a multitude of phenotypes - gastrointestinal, extraintestinal, subclinical, potential, seronegative, non-responsive, and refractory [3]. Although small intestinal biopsy is still being considered as the gold standard for the diagnosis of the disease, there is satisfactory upcoming evidence of serological autoantibodies such as anti-gliadin antibodies, anti-deamidated gliadin peptide antibodies, anti-tissue transglutaminase (anti-tTG) antibodies, and anti-endomysial (anti-EMA) antibodies in aiding in the screening of the disease as they have a superior margin of sensitivity and specificity [4]. There have been multiple approaches and modalities of treatment over the years, but a gluten-free diet is the only primary intervention that has successfully prevented the current manifestations as well as slowed the progression of the disease.

The insidious coexistence of endocrine diseases has been well documented and demonstrated in patients with CD. The common autoimmune background and a moderate overlap of symptoms and signs serve a grim prognosis and a stubborn outcome of treatment. There have been ample amount of studies that have illustrated the increased prevalence of endocrine conditions with a peculiar emphasis on type 1 diabetes mellitus (DM) and autoimmune thyroid conditions [5,6]. Other rare endocrine associations like adrenal insufficiency, hypoparathyroidism, infertility, and pituitary involvement have also been noted [7-10].

This article aims to:

1. Underline the growing concern of the endocrinopathies that are masked by the veil of CD due to the heterogeneity in its clinical presentation and the delay in the diagnosis of the disease itself.

2. Highlight the impact of CD on the endocrine system with an attempt to unravel the silent systemic autoimmune clinical impact of the coexistence of the disorders.

3. Vocalize the possibilities and advantages of screening, early diagnosis, and treatment for these conditions.

## Review

Methods

A robust search performed on databases including PubMed and PubMed Central was conducted where only peer-reviewed articles published from 1990 to 2020 in English language and relevant to the topic were included. The following are the inclusion/exclusion criteria that were applied:

1. Studies of all designs/types were included, and gray literature was excluded.

2. Although all age groups were included, a particular emphasis was placed on the pediatric and adult age group.

3. Studies performed globally irrespective of ethnicities and geography were included.

4. Studies done only on humans were included, and the rest were excluded.

Diabetes and CD

When it comes down to the basic pathophysiologic development of the disease, CD and type 1 DM share a common basis of autoimmunity where there is a development of antibodies that lead to the clinical progression of the diseases. Type 1 DM is a heterogeneous autoimmune disorder confined usually to the young population, where there are antibodies against the beta islet cells of the pancreas causing their destruction leading to a profound state of insulin deficiency and uncontrolled hyperglycemia. CD is one of the few autoimmune disorders, which has a very obscure primary trigger and is turned around with just avoidance of the causative agent. Over the course of time, the prevalence of coexistence of CD and type 1 DM has been well documented by numerous studies conducted across various parts of the globe [11,12]. There were several factors in motion as to why the incidence of CD and type 1 DM was lesser compared to what it is today. First, very little was known about both the diseases a long time ago in terms of their pathogenesis and trajectory of progression. Second, a distinct overlap in certain symptoms and signs such as diarrhea and weight loss that occurred in both the conditions could not be differentiated and narrowed upon. Currently, we stand on a very high pedestal in terms of the knowledge of the diseases, their respective screening processes, their individualized management, and also decoding of their non-classical presentations.

The genetic basis of the two diseases anchors itself on certain observations made such as the following:

1. Identical twins had almost a 50% and 75% chance of developing type 1 DM and CD, respectively, compared to the general population [5].

2. First-degree relatives had an increased risk of developing both the disorders compared to the general population [5,13].

The concept of linkage disequilibrium comes into play in this scenario where the sharing of HLA, specifically DR3 and DQ2, is evidently exhibited by both these conditions [14].

A study was conducted over a period of 19 years in Australia in a sample population of 4379 children below the age of 18 years, who were regularly followed up and subsequently categorized in different subsets with respect to their age. It was found that CD was diagnosed in diabetics at a percentage of 45, 78, and 95 at two, five, and 10 years of age, respectively. The study also concluded that children with type 1 DM onset at an age less than five years were at a greater risk of developing CD, thereby establishing a directly proportional relationship between the duration of insulin deficiency and that of gluten sensitivity [15] (Table 1).

**Table 1:** Summary of included studies linking celiac disease and type 1 diabetes mellitus. Anti-tTG, Anti-tissue transglutaminase; anti-EMA, anti-endomysial antibody; CD, Celiac disease; DM, diabetes mellitus.

References	Design	Cases of DM	Cases of CD	Population	Diagnostic criteria	Conclusion
Pham-short et al. (2012) [15]	Observational cohort study	4379	185	Children < 18 years from Australia	For CD – anti-tTG, anti-EMA antibodies, and small intestinal biopsy	CD was strongly associated with type 1 DM and was directly related to an earlier age of onset and a longer duration of the disease.
Saadah et al. (2012) [16]	Retrospective hospital record-based study	430	48	Children in Saudi Arabia	For CD – anti-tTG and small intestinal biopsy	Increased prevalence of CD in patients with type 1 DM
Salardi et al. (2008) [17]	Longitudinal cross-sectional study	331	22	Children in Italy	For CD – anti-EMA antibodies	Increased risk of CD in type 1 DM, which was triggered by environmental and infectious etiologies
Cerutti et al. (2004) [18]	Cohort study	4322	292	Children in Italy	For CD – anti-tTG, anti-EMA antibodies, and small intestinal biopsy	CD was strongly associated with type 1 DM and was directly related to an earlier age of onset and a slight female predominance.

In a retrospective hospital record-based cohort study performed on a group of children in Saudi Arabia, out of 430 cases of type 1 DM, 48 cases of CD were diagnosed. The study rested upon the conclusion that there was an increased prevalence of CD in patients with type 1 DM [16] (Table 1).

A peculiar finding was noted in a longitudinal cross-sectional study performed in Italy where it was discovered that patients underwent seroconversion with respect to CD after an average period of 18 months, suggesting a temporal relationship between the diseases and also raising thoughts about viral infections and environmental factors playing either an individualized or a combined role in serving as the trigger factor for both the conditions [17] (Table 1).

The findings of the above studies can be compared in parallel to another study that was conducted in Italy in the pediatric age group where 4322 diagnosed cases of DM were screened for the presence of CD. There were 292 cases of the biopsy-proven CD out of the entire sample population taken into consideration. This study too was conclusive of the fact that there was an increased prevalence of CD in patients with type 1 DM, and the coexistence of these diseases grew with an earlier onset of type 1 DM. Additionally, a noteworthy conclusion of a slightly higher female predominance was also made at the end of the study [18] (Table 1).

The co-occurrence of the two conditions warrants a strict screening protocol that has been implemented by the American Diabetes Association [19]. The screening is to be done by checking for the presence of

1. anti-tTG IgA antibodies,

2. anti-endomysial (anti-EMA) antibodies, and

3. anti-tTG IgG antibodies, if there is presence of IgA deficiency in the patient.

The guidelines are along the lines of the following [19]:

1. CD should be screened soon after type 1 DM is diagnosed and if negative to be followed up after two and five years.

2. Patients with type 1 DM exhibiting symptoms of CD should be screened as soon as possible irrespective of a previous negative result.

A multi-directional approach should be engineered toward the management of these two conditions keeping in mind the common background and the development of complications. Therefore, a gluten-free diet balanced by a properly tailored insulin regimen will help achieve a desirable blood glucose level and prevent malabsorption, thereby constructing an optimistic tangent to the prognosis of the patient.

CD and thyroid

When the thyroid gland is referred to with the context of autoimmunity, there are two prominent disorders that come up - Hashimoto's thyroiditis and Graves’ disease. Hashimoto's thyroiditis is a diversified autoimmune disorder where the presence of anti-thyroid peroxidase (anti-TPO) and anti-thyroglobulin antibodies dial the functioning of the thyroid gland causing transient hyperthyroidism and eventual hypothyroidism. Graves’ disease on the other hand is a type two hypersensitivity reaction where there are IgG thyroid-stimulating globulins, which lead to a state of thyrotoxicosis.

The eventual linkage between CD and autoimmune thyroiditis stems from certain hypothesized theories:

1. Similarly as in the case of type 1 DM, CD and autoimmune thyroiditis are linked by the fabric of HLA subtypes: HLA-DQ2, HLA-DR3, and B8. This varies largely between individuals and is more of a consequence of the multifactorial genetic inheritance [20,21].

2. Thyroid gland is differentiated from the pharyngeal gut sharing a common origin [21].

3. Increased penetration of the intestine leads to humongous amounts of precursor antigens in the bloodstream, which leads to a cross-reaction elsewhere in the body [22].

4. Tissue transglutaminase cross-reacts directly with the thyroid gland [23].

A cross-sectional screening study performed on a group of 12-year-old children from Sweden demonstrated a threefold increase in the presence of thyroid antibodies in cases diagnosed with CD [24] (Table 2).

**Table 2:** Summary of included studies linking celiac disease and thyroid diseases. Anti-tTG, anti-tissue transglutaminase; anti-EMA, anti-endomysial antibodies; CD, Celiac disease; DM, diabetes mellitus; FT4, FT3, free T4, free T3; TSH, thyroid-stimulating hormone; anti-ATG, anti-thymocyte globulin; anti-TPO antibodies, anti-thyroid peroxidase antibodies; anti-ARA, anti-reticulin antibodies.

References	Design	Cases	Control	Population	Diagnostic criteria	Conclusion
Van der Pals et al. (2014) [24]	Cross-sectional	335 cases of CD out of 12632 children	_	Children from Sweden	For CD – anti-tTG, anti-EMA antibodies, and small intestinal biopsy. For thyroid disease – anti-TPO antibodies	Increased prevalence of thyroid disease in patients with CD
Meloni et al. (2007) [25]	Case-control	324 cases of CD	8040, age matched	Children from Sardinia, Italy	For CD – small bowel biopsy. For thyroid disease – FT4, FT3, TSH, USG, and anti-TPO antibodies	Increased association of autoimmune thyroid conditions with CD
Kowalska et al. (2000) [26]	Case-control	34 cases of CD	28 with negative screening for CD	Children	For CD – anti-EMA antibodies. For thyroid disease – FT4, FT3, TSH, anti-ATG, and anti-TPO antibodies	Increased prevalence of thyroid antibodies in patients with CD
Velluzzi et al. (1998) [27]	Case-control	47 cases of CD	91 healthy controls	Adult population from Sardinia, Italy.	For CD – anti-tTG, anti-ARA antibodies, anti-EMA antibodies, and small intestinal biopsy. For thyroid disease – FT4, FT3, TSH, and anti-TPO antibodies	Increased prevalence of thyroid antibodies in patients with CD

A case-control study was performed in 2007 on a subgroup of children in Sardinia, Italy, where 8040 controls were considered alongside 324 cases of CD who were then subsequently screened, and there was an increased incidence of autoimmune thyroiditis in the patients with CD [25] (Table 2).

Another case-control study was performed in 2000 in a sample population of children where there were 34 cases matched with 28 controls. The study arrived at a conclusion of the presence of thyroid antibodies in the cases with a measure of 41.1% in comparison to the control group where the measure was 3.56% [26] (Table 2).

This could be aligned with a similar case-control study that was performed in 1998 on a subgroup of adult population in Sardinia, Italy, where 91 healthy controls were selected besides 47 cases having CD. The patients were screened and were found to have an increased prevalence of thyroid autoimmunity. A fascinating observation made in these patients was that of the presence of the DQB1*0502 genotype [27] (Table 2).

An evaluation of 111 patients with Graves’ disease at a thyroid clinic with healthy age-matched controls in the local population yielded an inference of the presence of CD in these patients of about 4.5% compared to that of 0.9% in healthy controls. An important highlight of this study was that the screening process leads to the diagnosis of three new cases of CD [28].

**Figure 1:**
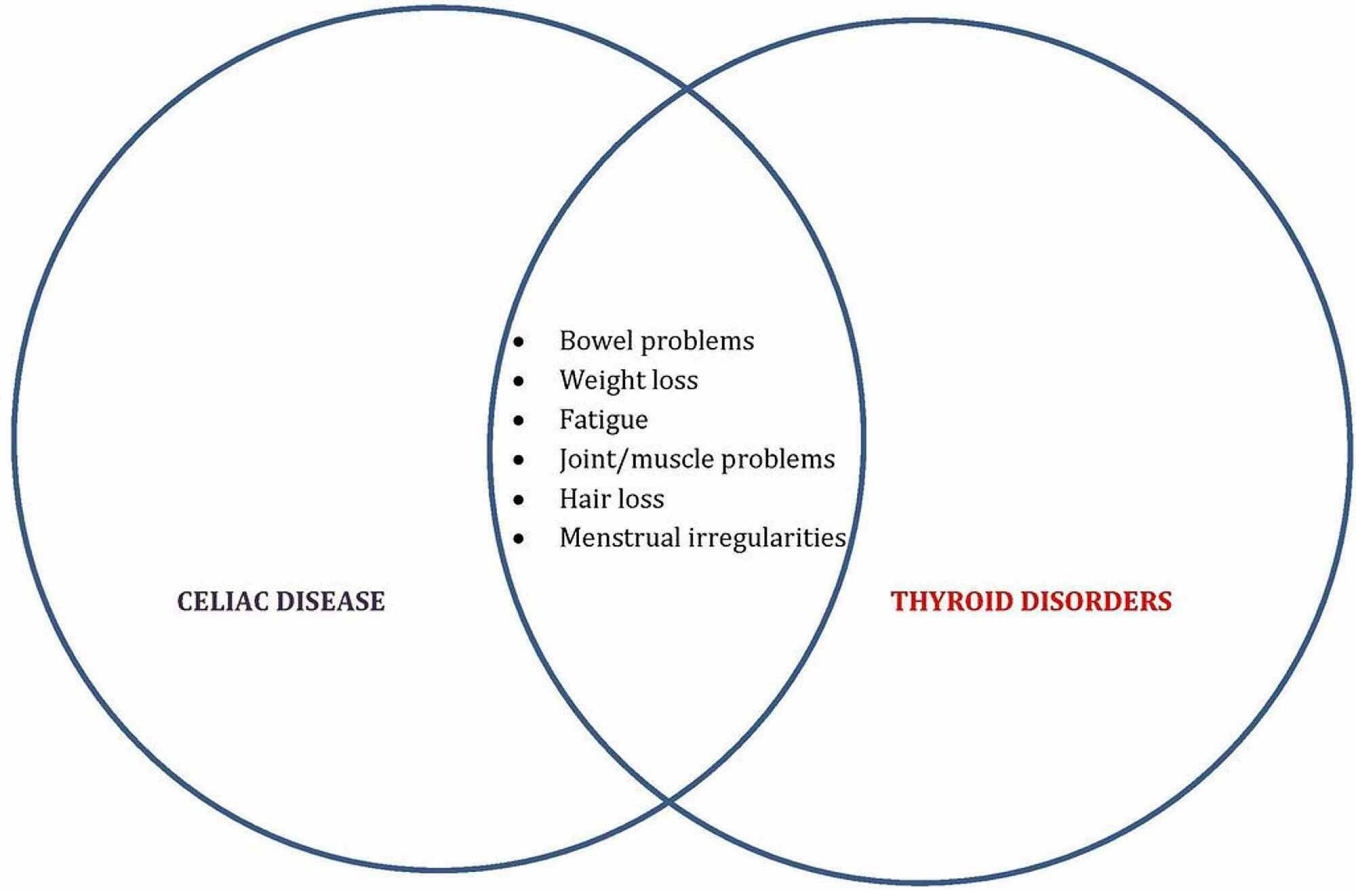
Overlap in the symptomatologies of Celiac disease and thyroid disorders.

The reason clinicians often arrive at crossroads along the course of diagnosis is due to the synonymous symptomology that occurs [21] (Figure 1):

1. The intestinal motility is a function of several hormones including the thyroid hormone, and hence diarrhea that occurs in CD can be variable depending upon the thyroid status.

2. Weight loss is a feature shared by CD as well as hyperthyroidism.

3. Malabsorption in CD can hinder the absorption of thyroid medications.

4. Menstrual cycle abnormalities can present themselves in rare cases in CD and is much more common in thyroid diseases.

5. Osteoporosis can present itself in CD and hyperthyroidism, both.

A very compelling rare occurrence of thyroid lymphoma as an extranodal complication of CD has also been recognized [29]. This in turn again reflects on the theory of the common embryologic origin of the thyroid and the primitive gut.

The point that has to be emphasized here is to screen all the patients with CD for the presence of thyroid autoimmunity and vice versa. A gluten-free diet has been argued considerably to single-handedly untangle the above-mentioned cobwebs created by the coexistence of these two diseases [30,31].

CD and other endocrine glands

Although the thyroid and the pancreas are the two most common glands involved in association with CD, there are very rare forms of endocrinopathies that have to be appreciated.

Adrenal insufficiency linked to CD has been reported in certain studies and case reports. A Swedish study was conducted where 14,366 cases of CD were identified through the national registry. After matching 70,095 references with the corresponding variables, it was found that there existed a temporal relationship between these two diseases, thereby testifying to the correlation between the two diseases [7]. Screening for CD is highly advised if a patient presents with adrenal insufficiency especially to the cases that are refractory to treatment.

The parathyroid gland works in close harmony with the gastrointestinal tract, kidneys, and bones to maintain adequate calcium balance in the body. Due to the villous atrophy that occurs in CD, there is decreased absorption of calcium and vitamin D along the gastrointestinal tract, which naturally leads to decreased calcium levels in the serum and therefore leading to secondary hyperparathyroidism. After a long deceitful period, the bone gradually loses the calcium and eventually weakens over time leading to osteoporosis due to which the patient presents with symptoms of bone pain, fatigue, etc. In a study conducted with 66 cases of CD and 76 healthy controls, patients with a longer duration of CD were more vulnerable to osteoporosis compared to the ones that were diagnosed earlier and the controls. The study also deduced that a gluten-free diet was necessary for children to achieve adequate bone mineralization [32].

Although as explained above, secondary hyperparathyroidism is relatively a common occurrence among patients with CD. Hypoparathyroidism is a much more isolated disease that occurs in relation to CD. There have been case reports suggesting the coexistence of these two conditions based on their customary autoimmune framework, but improvement was noted on adherence to a gluten-free diet [8,33].

Development of antibodies against the pituitary gland was well demonstrated in a cross-sectional study that was conducted in Italy, in which out of 119 patients diagnosed with CD, 50 patients were found to have pituitary antibodies as further investigations unfurled [10]. The implications of the resultant damage to the pituitary gland could be catastrophic in terms of stunted growth, hypogonadism, and prolactin imbalances.

Ovarian failure and infertility can sometimes be masked by the blanket of subclinical CD resulting in menstrual irregularities and premature menopause. There have been studies that have very clearly harbored a correlation between these two conditions with infertility affecting not only women but also men [34,35].

## Conclusions

Despite CD presenting with predominant gastrointestinal involvement, there is a very complex and diverse extraintestinal involvement that can make the diagnosis cumbersome. The main point to be highlighted in this article is to see the CD from a multi-system perspective and orchestrate a multicentered approach toward the disease rather than just looking at it from one standpoint. As this article primarily focused on the endocrine involvement, it is important to realize that the limited prevalence of the simultaneous presence of these disorders leads to restricted documentation of these illnesses leaving most aspects of these diseases undiagnosed. This can be rectified by a stringent screening protocol along with wide availability of screening tools, and widespread awareness of these silent yet easily treatable disorders will propel the patient into a land of promising recovery and a long-term favorable prognosis.
